# Medical and Surgical Uses of Spirits of Turpentine

**Published:** 1885-02-20

**Authors:** J. McF. Gaston

**Affiliations:** Professor of Principles and Practice of Surgery in the Southern Medical College, Atlanta, Georgia


THE
### Southern Medical Record.

EDITORS:
THOMAS S. POWELL, M.D.  R. C. WORD, M.D.

*R. C. WORD, M.D., Managing Editor.*

☞All Communications and Letters on Business connected with the RECORD must be addressed to the Managing Editor.

VOL. XV. ATLANTA, GA., FEBRUARY 20, 1885. No. 2.

### ORIGINAL AND SELECTED ARTICLES.

### MEDICAL AND SURGICAL USES OF SPIRITS OF TURPENTINE.

By J. McF. GASTON, M. D.,
Professor of Principles and Practice of Surgery in the SOUTHERN MEDICAL COLLEGE, Atlanta, Georgia.

Having observed frequent allusions latterly to the employment of the spirits of turpentine for its general and local effects upon the organism, it may not prove unacceptable to present some data from my experience with this article in medical and surgical cases during a series of years.

It is canonized as a domestic remedy throughout the Southern States; and we rarely find the voice of the people generally expressed in such strong terms in regard to a medicinal agent as it comes forth from every class of the community in favor of the external and internal use of the spirits of turpentine, without having an underlying basis of trustworthy results. We should always have great respect for the disinterested expression of a public opinion by the laity in matters of fact connected with their practical and commonplace uses of medicines. This is something very different from the trumped-up praises of a proprietary remedy; and while quackery seeks to bolster up its nostrums by flaming advertisements that are flaunted in the faces of every passer-by, we hear and see the fruits of every-day observation among the masses favorable to this domestic catholicon.

Since I listened long, long ago to the teachings of Dr. George B. Wood in the University of Pennsylvania respecting the virtues of the internal administration of the spirits of turpentine in enteric fever, and other intestinal and peritoneal troubles, my conviction of its superior efficacy has been strengthened by the application of this mode of treatment, not only in these affections, but in many other disorders of the serous and mucous membranes. Commencing with the mouth, its corrective powers have been tested throughout the entirety of the tract of the alimentary canal, and likewise in the renal and vesical conduits, so that no agent has precedence to it in the functional disturbances of these several organs.

The great bugbear of Bright's disease, which is now coming to be recognized rather as a derangement of the chylopætic viscera than a proper kidney disease, has not been modified more satisfactorily by any other medication than by the moderate and continued employment of this article. It is to be hoped that the mode of studying diseases by their effects, as in the observation of albuminous urine in this grave disorder, may give place to a more rational and philosophic search for the causes of the derangement in the physical organization, and that remedies may be directed to the correction of the sources of troubles in the animal economy.

If we should undertake to locate the spirits of turpentine in the classification of remedies in the Materia Medica, it would occupy most fitly a place under nearly every heading, according to the variety in the modes of its use, and be entitled to rank with the most approved medicaments in the list of internal and external agencies so classified in therapeutics. This is no unmeaning claim, based upon an extravagant conception of its wide range of medical properties on theoretic principles, but the sober and serious statement of positive knowledge of its effects, from actual application in a great variety of disorders.

We would note its recognized properties as a purgative in doses of a tablespoonful, as a diuretic in teaspoonful doses, as a nervine and anodyne when given in portions of fifteen or twenty drops at frequent intervals, as an alterant and aseptic remedy in ten drop doses repeatedly, while its corrective powers for indigestion when taken occasionally in the dose of five drops has made it a standby in some families. To recount the long train of ills to which the spirits of turpentine is found useful in the domestic practice of thousands of families would carry me far beyond the limits which can be allowed in this brief notice. But it is not only the old women who, at the present day, discover the charms of this agent as a local application, as many of the most generally employed liniments which have the most extensive sale and find the greatest number of recommendations from their consumers, contain spirits of turpentine. The medical profession is united in giving it the pre-eminence for sprains and bruises, for aches and swellings of the joints, and for mangled wounds which might lead to gangrene or tetanus. Within my sphere of observation, there is no combination of medicinal agents equal to spirits of turpentine with camphor for averting the bad effects of local injuries to the superficial or deep-seated tissues, and parts which are ecchymosed, or as styled in common parlance, blood-shotten, from blows or by falls, are relieved within a few hours by an application of this liniment upon pieces of flannel bound over the discolored spot.

But it is not to these ordinary uses of the article that the attention of the medical profession is now called. It has been brought to the notice of practitioners of medicine and surgery as a valuable injection into the parenchymotous structure of malignant tumors, and for inhalation of its vapors in diphtheria, in a way to give it an importance as a special medication in this class of cases. It has been resorted to as a disinfectant with the most satisfactory results, and there is not in the whole list of aseptic agents anything so powerful in arresting gangrene in the adynamic disintegration of tissues which accompanies extensive injuries. The internal and external use of the spirits of turpentine in cases of peritonitis is unequalled, for the arrest of this serious form of inflammation in the abdomen. Nothing is known to compare with this article, mixed in castor oil, for colic and intestinal obstructions; while it is our sheet-anchor after a dose of calomel in biliary troubles. But its use after more active treatment in suppression of the secretion of bile or in subacute hepatitis has not received that consideration from physicians to which my observation of its salutary influence would entitle it, and I have to urge upon those having obstinate cases of hepatic torpor a resort to this means of relief.

As an emmenagogue, likewise, I have secured most satisfactory effects from the spirits of turpentine, and in uterine catarrh it has given better results than other measures of treatment, while in leucorrhœa and in gonorrhœa I have employed it to advantage in conjunction with other medicines.

There is a species of atonia of the pelvic viscera that predisposes to displacement of the womb, in which the small doses of spirits turpentine kept up for several weeks has proved very beneficial in my hands.

It has been found the most efficient remedy for diphtheria when administered internally as well as by its local application to the fauces and the inhalation of its fumes when burnt with tar, as already stated, and my favorable results in the management of this usually serious degeneration of the elementary structures is due to the thorough impregnation of the system with this antidote to blood-poisoning. If septicæmia is capable of being arrested in any case, I would rely more upon the saturation of the secretions with this pervading depurient than upon any other agent.

In hemorrhagic disorders, the spirits of turpentine has been used with great advantages internally and locally, and though I have no personal experience in the hematuria and intestinal hemorrhages of typho-malarial fever, it has been used by others with benefit in this relaxation of the mucous surfaces. It is known as a valuable hemostatic by all surgeons who have had occasion to use it for the oozing which is sometimes so troublesome from extensive surfaces after operations, and has been attended with prompt relief by topical use in uterine hemorrhage. In that state of things following the retention of the placenta, and in offensive lochial discharges, there is no better corrective than swabbing out the uterine cavity with undiluted spirits of turpentine.

There is evidently a tolerance of these local applications to the mucous membrane and to the cutaneous surface in man which does not exist in the inferior animals; and the only drawback to the general employment of the spirits of turpentine internally is its occasianal irritation of the urinary organs, inducing strangury.

This, however, may be obviated by combining camphor with it, and amongst the many patients to whom spirits of turpentine has been given, I have only met with two individuals with idiosyncracies which precluded its use.

I have to prefer a claim for its recognition by the medical profession among the elegant preparations which grace the shelves of the pharmaceutist and apothecary of this progressive age. In the form of capsules, as prepared by Clertan, the spirits of turpentine can be taken by persons who have a repugnance to its taste and smell; and I have requested some of our druggists to keep it in this form, so that it is supplied to my patients thus whenever prescribed. In these capsules the French use the spirits of turpentine extensively in neuralgias, and insist that it is the most efficacious treatment for this painful affection. My individual observation of its application to this disorder does not corroborate this high estimate of its curative agency, yet I have employed it in neuralgia with benefit.

There is a complication of pectoral with enteric disease, known generally as typhoid pneumonia, in which a combination of the spirits of turpentine with camphor and carbonate of ammonia has acted magically in a considerable number of cases, and I rely upon this mixture very much in typhoid fever, uncomplicated with the pneumonic symptoms. The spirits of turpentine forms the basis of a cough syrup which has been attended with benefit in chronic catarrhal affections; and, indeed, it works well in all derangements of the mucous membranes, as well as in most of those involving the serous membranes.

It is owing to its property of stimulating the capillaries that it serves a good purpose so frequently as a surgical dressing, and its antiseptic qualities cause it to arrest any degeneration in the adjacent tissues when applied to punctured wounds, even correcting the dangers resulting from dissection wounds or other septic contamination.

